# Analysis of Electromagnetic Characteristics of Copper-Steel Composite Quadrupole Rail

**DOI:** 10.3390/ma15175851

**Published:** 2022-08-25

**Authors:** Tengda Li, Gang Feng, Chong Du, Pengxiang Zhang

**Affiliations:** Air and Missile Defense College, Air Force Engineering University, Xi’an 710051, China

**Keywords:** electromagnetic launch, composite material, composite layer, rail, electromagnetic characteristic

## Abstract

The ablation and wear of the four-rail electromagnetic launcher during the working process will aggravate the damage of the armature and rail, and greatly affect the service life of the launcher. To effectively alleviate rail damage, this paper applies the copper-steel composite rail to the four-rail electromagnetic launcher and proposes a new four-rail electromagnetic launcher. Based on the quadrupole magnetic field theory, the physical model of the new four-rail electromagnetic launcher is established, and the electromagnetic characteristics of the ordinary and new launchers are compared and analyzed using the finite element method. On this basis, the influence of composite layer parameters on the electromagnetic characteristics of copper-steel composite quadrupole rail is explored. The study found that the new four-rail electromagnetic launcher can provide a better launch magnetic field environment for smart loads, and the current distribution of the armature and the rail contact surface is more uniform, which can effectively improve the contact condition between the armature and the rail. The composite layer parameters of copper-based composite rail will have a certain impact on electromagnetic characteristics, and copper-steel composite rail of appropriate proportions can be selected according to different needs. The model proposed in this paper has a certain degree of scientificity and rationality.

## 1. Introduction

The electromagnetic launch is a new weapon launch technology that uses electromagnetic thrust to accelerate the load to ultra-high speed [1], with the advantages of great power, strong concealment, and controllable thrust [2,3], and has attracted great attention from all over the world [4,5]. As the technology continues to evolve, the need to launch new smart carriers such as missiles, aircraft, and satellites becomes increasingly urgent [6,7,8], but these loads contain a large number of precision electronics that are extremely sensitive to the magnetic field environment of the launch. The four-rail electromagnetic launcher (FREL) can effectively achieve magnetic field shielding and better meet the requirements of launching new smart carriers, and has broad development prospects [9,10,11].

However, the proposal of FREL has not yet been able to solve the problem of ablation wear of rail [12], which greatly restricts the development of FREL. As one of the important components of the device, the inner side of rail faces ablation, wear and other problems, and its conductivity, stiffness, and other characteristic requirements are high. The comprehensive performance of rail to a large extent determines the actual application of the launcher, so the design of the rail and the choice of materials has become the key to solving the problem. The composite materials can take into account all aspects of performance, the proposal and application of composite rails provide new ideas for solving the above problems [13]. The composite rail refers to the use of composite material designability, according to the performance requirements and use needs, through the material composition and configuration design of the corresponding rail, due to the good conductivity of copper materials, the rail mostly uses copper-based composite rail [14,15].

To prolong the life of rail, improve the launch accuracy, and alleviate damage during the launch, a new type of copper-based composite rail can be used, which has been studied a lot by scholars at home and abroad in recent years [16,17]. Cao [18] experimentally studied the thermal ablation characteristics of copper diamond electromagnetic rails in the initial stage of launch, and found that they are closely related to current and preload. Barbara [19] investigated the degree of damage of different material orbitals at different launch energies, and found that the Cr/Cu composite orbits had less damage at low energies. An XY [20] analyzed the contact stresses on the copper-based composite rail and armature contact surfaces, and found that the stress caused by the temperature rise of the armature had a greater impact on the rail. The above research is based on ordinary rail electromagnetic launchers, and the application of copper-based composite rail on FREL has not been explored.

In this paper, the electromagnetic characteristics of copper-steel composite rail in the new four-rail electromagnetic launcher (NFREL) are mainly studied, and the model of NFREL is established, and the electromagnetic characteristics of the new and ordinary FREL are compared and analyzed using the finite element method. The influence of the geometric parameters of the composite layer on the electromagnetic characteristics is discussed, the rationality and superiority of the copper-steel composite quadrupole electromagnetic rail are verified, and the theoretical reference is provided for the design and application of the composite four-rail electromagnetic launcher.

## 2. Quadrupole Magnetic Field Theory

**Figure 1:**
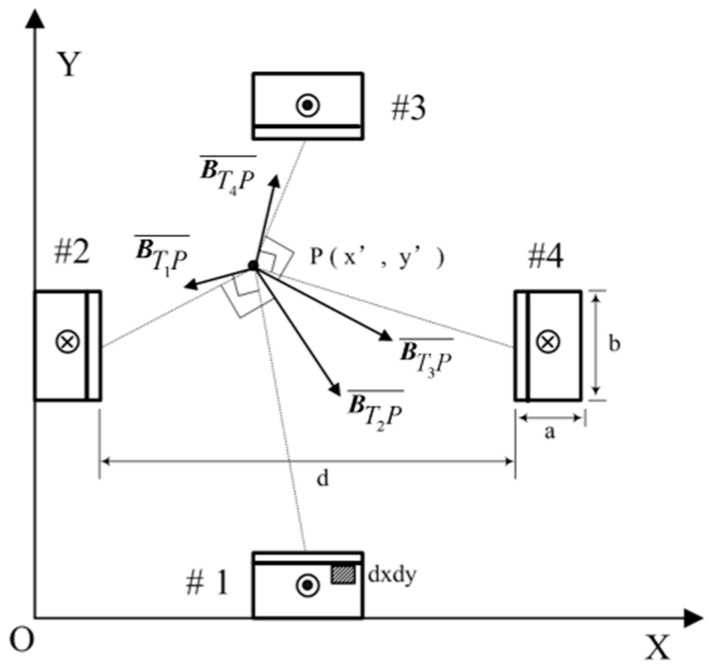
Schematic of the magnetic field generated by the rail current.

As shown in Figure 1, a certain launch cross-section is selected to analyze the magnetic field strength generated by the rail current in the launch region.

The rails are numbered m=1,2,3,4 sequentially, with the launcher aperture d, the composite rail width a, the rail height b, and the current isar density J, according to the Biot-Savart Law:

(1) $$dB=\frac{\mu_{0}}{4\pi }\frac{Idl\times R}{\left|R^{3}\right|}$$

The magnetic field strength generated by the cross-sectional current source Jdxdyk at the point $P(x^{\prime },y^{\prime })$ in rail 1 is

(2) $$d\bar{B_{T_{1}P}}=\frac{\mu_{0}J}{4\pi }\frac{dxdy}{\left|R^{3}\right|}\left[k\times \left(x-x^{\prime }\right)i+k\times \left(y-y^{\prime }\right)j\right]$$

where $\mu_{0}$ is the permeability in the vacuum, k is the unit vector of the direction of the rail current, R is the distance vector between the center point of the current source (x,y) and the $P(x^{\prime },y^{\prime })$, i.e., $R=(x-x^{\prime })i+(y-y^{\prime })j$, then $\bar{B_{T_{m}P}}$ is the magnetic field strength generated by the current of the *m*th rail section at the point $P(x^{\prime },y^{\prime })$, and the current in the section of the rail 1 can be integrated to obtain the magnetic field strength in the section of the rail

(3) $$\bar{B_{T_{1}P}}=\underset{s_{1}}{∯}\frac{\mu_{0}J}{4\pi \left|R^{3}\right|}\left[k\times (x-x^{\prime })i+k\times (y-y^{\prime })j\right]dxdy$$

where $S_{1}$ is the cross-sectional area of the copper rail of the first composite rail.

Then the magnetic field strength excited by the current in the section of the *m*th composite rail at point $P(x^{\prime },y^{\prime })$ is

(4) $$\bar{B_{T_{m}P}}=\underset{s_{m}}{∯}\frac{\mu_{0}J{\left(-1\right)}^{m+1}}{4\pi \left|R^{3}\right|}\left[k\times \left(x-x^{\prime }\right)i+k\times \left(y-y^{\prime }\right)j\right]dxdy$$

Extending the above results to three-dimensional space, the magnetic field strength of the *m*th composite rail section current in the spatial region is

(5) $${\bar{B_{T_{m}P}}}^{\prime }=\underset{S_{m}}{∯}\frac{\mu_{0}J}{4\pi }\frac{{\left(-1\right)}^{m+1}}{\left|R_{1}{}^{3}\right|}\left[k\times \left(x-x^{\prime }\right)i+k\times \left(y-y^{\prime }\right)j+k\times \left(z-z^{\prime }\right)k\right]dxdy$$

where $R_{1}=(x-x^{\prime })i+(y-y^{\prime })j+\left(z-z^{\prime }\right)k$.

When the armature moves along the rail to z(t), the magnetic field strength of the *m*th rail energized segment in the space region is

(6) $$\begin{array}{l} B_{T_{m}P}=\int_{0}^{z\left(t\right)}{\bar{B_{T_{m}P}}}^{\prime }dz=\\ \int_{0}^{z\left(t\right)}\left[\underset{S_{m}}{∯}\frac{\mu_{0}J}{4\pi }\frac{{\left(-1\right)}^{m+1}}{\left|R_{1}{}^{3}\right|}\left[k\times \left(x-x^{\prime }\right)i+k\times \left(y-y^{\prime }\right)j+k\times \left(z-z^{\prime }\right)k\right]dxdy\right]dz \end{array}$$

According to the vector superposition principle of the magnetic field, the electromagnetic field strength generated by the four composite rails at $P(x^{\prime },y^{\prime },z^{\prime })$ is

(7) $$B_{TP}=\sum_{m=1}^{4}B_{T_{m}P}$$

**Figure 2:**
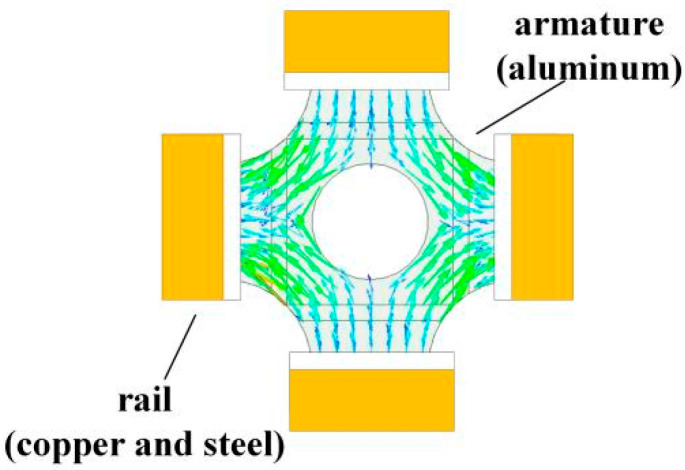
Armature current density vector diagram.

The current distribution of the armature is shown in Figure 2, and it can be seen that the current is mainly concentrated at the four-stage drainage arc [21], and the magnetic field strength generated by the current in the armature in the launch area $P\left(x^{\prime },y^{\prime },z^{\prime }\right)$ is

(8) $$B_{AP}=\sum_{n=1}^{4}B_{A_{n}P}=\frac{\mu_{0}}{8\pi }\sum_{n=1}^{4}\int_{l_{n}}\frac{Idl_{n}\times R^{\prime }}{\left|{R^{\prime }}^{3}\right|}$$

where $B_{A_{n}P}$ is the magnetic field strength generated by the *n*th stage drainage arc at $P\left(x^{\prime },y^{\prime },z^{\prime }\right)$; $l_{n}$ is the length of the *n*th-segment drainage arc, $R^{\prime }=\left(x-x^{\prime }\right)i+\left(y-y^{\prime }\right)j+\left(z(t)-z^{\prime }\right)k$.

The magnetic field strength at any point in space is the sum of the magnetic field strength generated by the rail and the armature is

(9) $$\begin{array}{l} B_{p}=B_{AP}+B_{TP}\\ \;\;\;\;=\frac{\mu_{0}}{8\pi }\sum_{n=1}^{4}\int_{l_{n}}\frac{Idl_{n}\times R^{\prime }}{\left|{R^{\prime }}^{3}\right|}\\ \;\;\;\;\;+\sum_{m=1}^{4}\int_{0}^{z\left(t\right)}\left[\underset{S_{m}}{∯}\frac{\mu_{0}J}{4\pi }\frac{{\left(-1\right)}^{m+1}}{\left|R_{1}{}^{3}\right|}\left[k\times \left(x-x^{\prime }\right)i+k\times \left(y-y^{\prime }\right)j+k\times \left(z-z^{\prime }\right)k\right]dxdy\right]dz \end{array}$$

## 3. Model Establishment and Condition Setting

### 3.1. Physical Model of FREL

**Figure 3:**
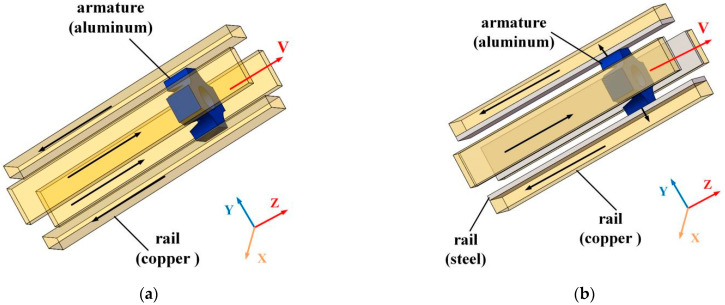
The model of two launchers.(**a**) The model of FREL; (**b**) The model of NFREL.

The model of FREL and NFREL is shown in Figure 3.

In the NFREL, the rail is based on copper with good electrical and thermal conductivity as the base material, which can ensure the flow-through capacity and the magnetic field environment required for launch. Steel with good stiffness and ablation resistance as a reinforcing material can improve the wear resistance of the rail. The current flows in from the copper rail end surface of the composite rail, and then flows through the rail path through the armature from the adjacent rail. The current of the rail creates a quadrupole magnetic field in the launch region, which is orthogonal with the current flowing through the armature to push the armature to move in the +z direction. The copper-based rails are used to generate and conduct currents, and provide magnetic fields, and steel rails are used to carry armature.

The caliber of NFREL is 80 mm×80 mm, the length of the copper rail is 1000 mm, the height is 40 mm, and the width is 18 mm; The length l and height h of the steel rail are consistent with the copper rail; The length of the armature is 40 mm, and the thickness of the throat is 15 mm. The caliber, rail length, rail height and width of the FREL are consistent with the new four-rail electromagnetic launcher; The rail and armature material properties are shown in Table 1.

### 3.2. Simulation Conditions and Method

**Figure 4:**
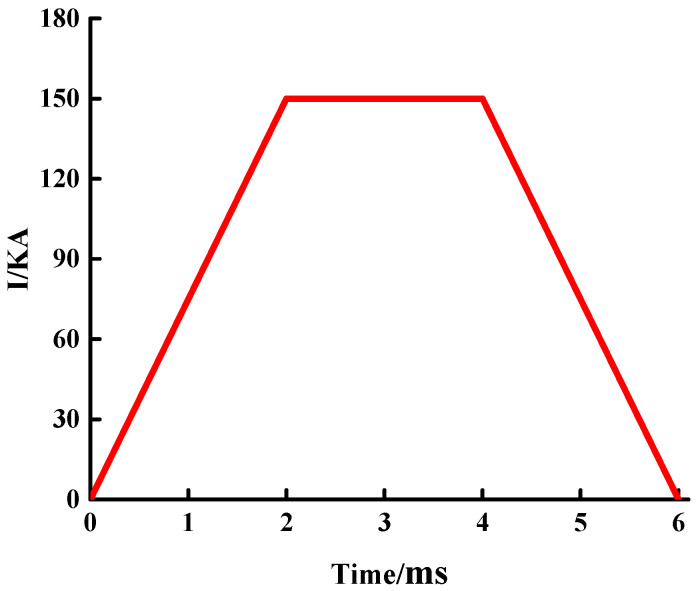
Transient simulation current diagram.

After the two models are established, the relevant functions need to be built to load the current. Electromagnetic launch is a complex transient process that should be simulated using transient currents. Through the waveform research, it is found that the direct application of the pulsed strong current on the rail will generate a relatively strong electromagnetic force, and the trapezoidal current waveform has the best effect. Therefore, the trapezoidal excitation is selected for the transient simulation current used in this section, as shown in Figure 4. The entire current conduction time is 6 ms, the peak value is 150 KA, and the current peak duration is 2 ms. Consider the current skin effect and set the vacuum region of the solution to 300%.

When using different size meshes to divide the model, the calculation time and computer resources required are different, and the calculation results are also different. Therefore, the accuracy of the calculation results under different size meshing needs to be verified. The maximum grid size of the armature in the control model is 0.5 mm, 1 mm and 2 mm respectively, and the maximum current density of the rail is calculated, and the result is shown in Table 2.

Comparing the maximum current density obtained by solving with different grid sizes, it can be seen that when the maximum grid size is 1 mm, the maximum current density of the armature is $7.28\times 10^{9}\;A/m^{2}$; When the maximum grid size is 0.5 mm, the maximum current density is $7.48\times 10^{9}\;A/m^{2}$, and the error of the two calculation results does not exceed 2.7%, indicating that the grid division is reasonable. To improve the calculation efficiency, the maximum mesh size is 1 mm to divide the mesh, and the armature and the rail contact are refined, and the maximum mesh size at this place does not exceed 0.5 mm.

In this paper, we propose using the finite element method to simulate the model of FREL and NFREL. The finite element method, also known as the matrix approximation method, is based on the variational principle and the weighted margin method. The basic idea is to simplify the solution domain of a complex system problem to a large number of finite interconnected non-superimposed subdomains, by solving the solution of the subdomain and then using the variational principle or the weighted margin method to derive the approximate solution of the entire system, using this method to achieve high-precision approximation calculation of the simulation model of this paper.

## 4. Analysis of the Simulation Results

### 4.1. Analysis of the Current Density Distribution

The distribution of the current density has an important influence on the magnetic field distribution and the location of the heat production. The site where the current density accumulates has a high heat production, which is easy to cause thermal damage to the rail and further affects the life of launcher. Therefore, the analysis of the current density distribution is extremely important. For the convenience of description, FREL can be described as ordinary type, and NFREL can be described as new type.

**Figure 5:**
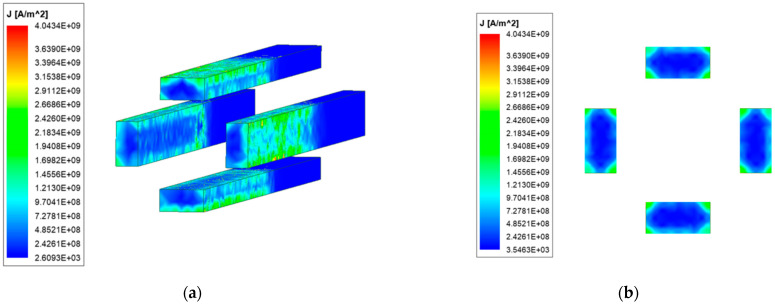
Rail current distribution of ordinary type. (**a**) Current density distribution; (**b**) Current distribution of the rail cross-section.

**Figure 6:**
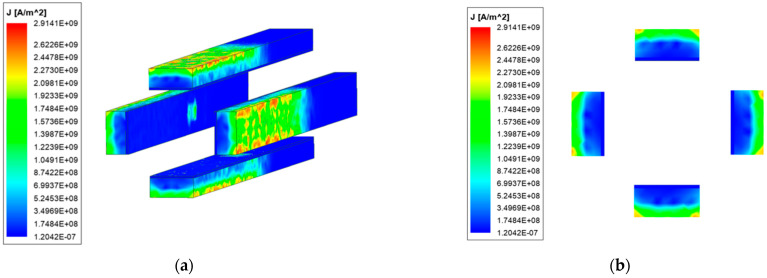
Rail current distribution of new type. (**a**) Current density distribution; (**b**) Current distribution of the rail cross-section.

Figure 5 and Figure 6 are the current density distribution of ordinary quadrupole rail and copper-steel composite quadrupole rail at 4 ms, respectively.

From Figure 5 and Figure 6, it can be seen that the current density distribution of ordinary and copper-steel composite quadrupole rail is different. The current density of ordinary quadrupole rail is reached $4.04\times 10^{9}\;A/m^{2}$, while the copper-steel composite quadrupole rails is only $2.91\times 10^{9}\;A/m^{2}$. From the perspective of the location of the current distribution, the difference is also more obvious: the ordinary quadrupole rail current is distributed very little in the middle area, and mainly distributed in the surface thin layer of the rail and the four edges, which is caused by the skin effect and proximity effect of the current: the two adjacent railcurrent directions are opposite, which meets the conditions of the current proximity effect, so the currents are close to each other, the distribution of the current on the inner edge is more concentrated, indicating that the proximity effect is also a factor that must be considered in electromagnetic analysis. The composite rail current is mainly distributed on the copper rail. There is no obvious current concentration in the inner edges and corners of the copper-based rail. The current flows centrally at the junction of the armature and the rail, so there is a large current density at this location. 

Although the geometry of the two rails is the same, the current area of the copper-steel composite quadrupole rail is smaller than that of the ordinary rail, and after loading the same size current, the maximum current density of the ordinary quadrupole rail is greater than that of the copper-steel composite quadrupole rail, which may be because the skin effect and proximity effect of the current in the ordinary quadrupole rail are more obvious, and the current is more concentrated on the edge, while the copper-steel composite quadrupole rail has a large current density only at the contact due to the characteristics of the rail material. It can be seen that the copper-steel composite rail can reduce the maximum current density of the rail and alleviate thermal damage to a certain extent.

**Figure 7:**
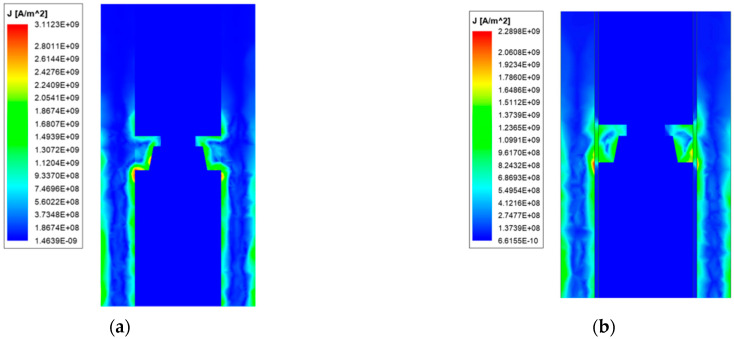
Axial current distribution of the launcher (**a**) Ordinary type; (**b**) New type.

To more clearly explore the distribution of current in the rail, the cross-section shown in Figure 7 is selected for analysis.

As can be seen from Figure 7, the current of the ordinary type is concentrated on both sides of the rail, and there is almost no current distribution in the middle area of the rail. The current is mainly conducted from the side of the armature. When the current flows to the contact of the rail and armature, part of the current flows in from the tail of the armature due to the short-circuit effect of the current.

Because the resistivity of the copper rail is the smallest, followed by the aluminum armature, and the resistivity of the steel rail is the largest, part of the current will accumulate at the front of the armature contact interface and flow from the armature head. The current in the copper-steel composite quadrupole rail is more distributed in the copper rail, and the current flows into the armature through the steel rail at the contact. It can be seen that the current of the copper-steel composite rail flows into the armature more evenly, which is quite different from the ordinary rail. It shows that the structure of the new type can alleviate the unevenness of the current distribution of the contact surface.

**Figure 8:**
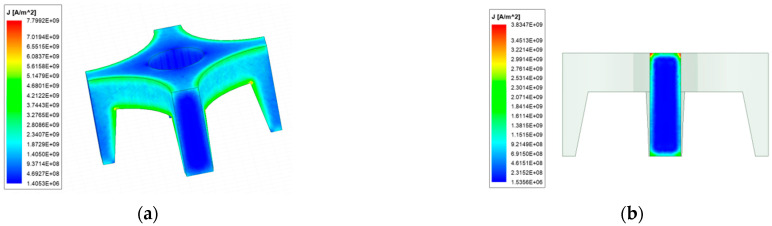
Armature current distribution of ordinary type. (**a**) Current distribution; (**b**) Current distribution of the contact surface.

**Figure 9:**
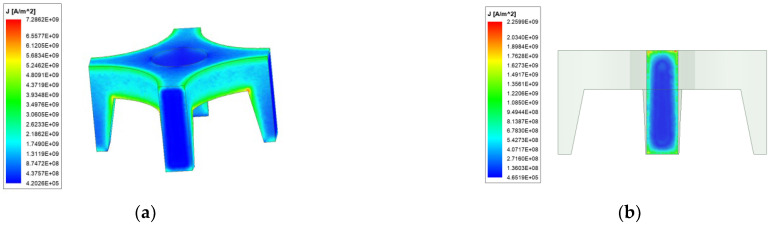
Armature current distribution of new type. (**a**) Current distribution; (**b**) Current distribution of the contact surface.

To more intuitively reflect the distribution of the current, the armature and the contact surface are simulated for analysis, and the results are shown in Figure 8 and Figure 9.

Comparing Figure 8a and Figure 9a, it can be seen that due to the special structure of the armature, the current is mainly distributed on the four drainage arcs, indicating that the design of the drainage arc is reasonable and can play a role in centralized conduction of current. There is also a large current distribution on the edge of the inner edge of the armature arm, and the concentration at the throat of the armature is severe, which is determined by the shortest path of the current. Analysis of Figure 8b and Figure 9b shows that since the rail width is larger than the armature width, the current also flows into the armature from both sides of the armature arm. Affected by the proximity effect of the current, the current around the contact surface is relatively concentrated, and there is almost no current distribution in the middle of the contact surface. However, the zero-area current area of contact surface of the new type is smaller than that of the ordinary type, and the maximum current density is mainly concentrated in the head and tail of the contact surface.

In terms of current size, the composite rail can reduce the maximum current density on the contact surface, and the zero area of the contact surface is larger, which can improve the current distribution on the contact surface.

**Figure 10:**
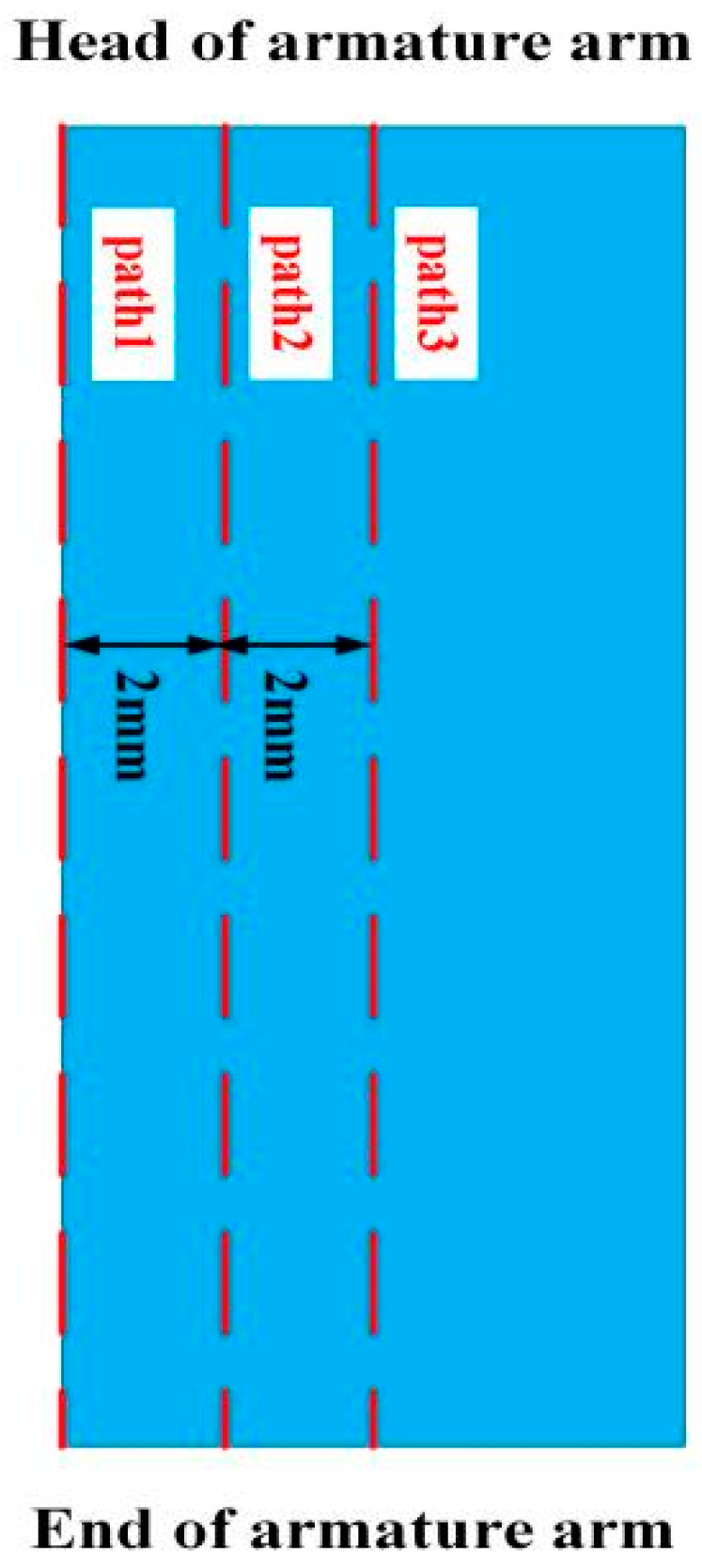
Schematic diagram of the path.

**Figure 11:**
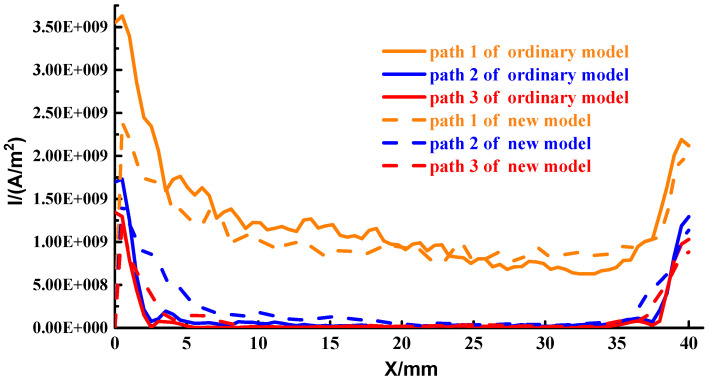
Current distribution diagram in the three paths.

To quantify the current distribution of the contact surface, we select path 1~path 3 as shown in Figure 10. Extract the magnitude of the current density on each path, as shown in Figure 11. The abscissa in the figure represents the distance from the head of the armature, and the ordinate coordinate is the current density.

As can be seen from Figure 11, the current distribution changes across paths are consistent. The difference in the current density of the head and tail on path 1 of the ordinary type is $1.7\times 10^{9}\;A/m^{2}$, and the difference of the new type is $0.533\times 10^{9}\;A/m^{2}$, it can be seen that the current density distribution of the contact surface of the new type is more uniform, which is consistent with the previous analysis, which can more effectively alleviate the thermal damage of the contact surface. The distribution of paths 2 and 3 is consistent with path 1, except that the current density in the middle of paths 2 and 3 is almost 0, i.e., there is no current distribution. It is explained that the current on the contact surface is mainly distributed in a limited area on both sides of the contact surface.

### 4.2. Analysis of the Magnetic Field Strength Distribution

**Figure 12:**
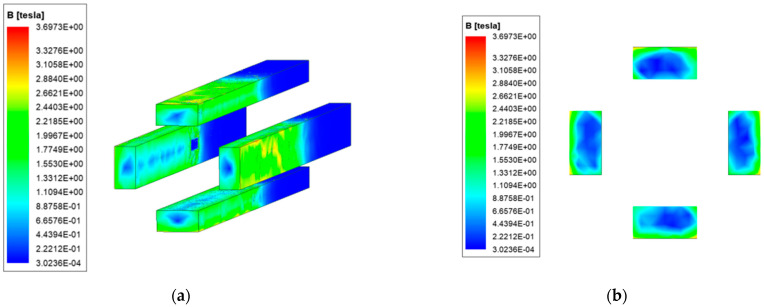
Ordinary type. (**a**) Magnetic field strength distribution; (**b**) Magnetic field strength distribution of the rail cross-section.

**Figure 13:**
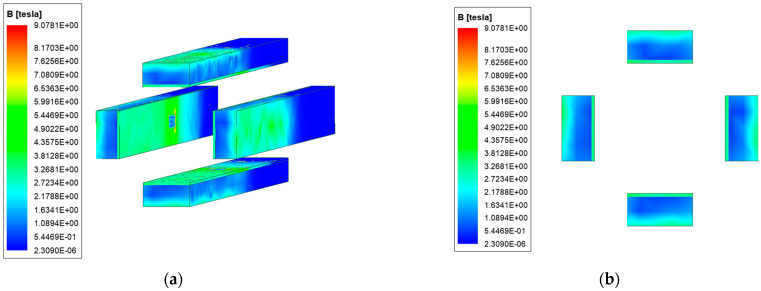
New type. (**a**) Magnetic field strength distribution; (**b**) Magnetic field strength distribution of the rail cross-section.

The magnetic field strength distribution is affected by the current distribution and can affect the operating performance of the electronic device of the load. On the basis of the analysis in the previous section, the magnetic field strength of ordinary and new type is analyzed. The results are shown in Figure 12 and Figure 13.

Due to the distribution of current, the magnetic field strength is also mainly concentrated on the surface of the rail, and the edge of the ordinary quadrupole rail is the most obvious. The magnetic field strength distribution of copper-steel composite quadrupole rail is similar to that of ordinary type, but with one obvious feature: there is a concentration of magnetic field strengths at the copper-steel rail interface. 

**Figure 14:**
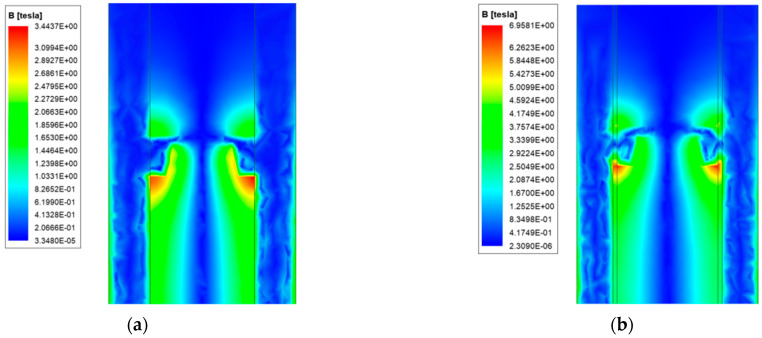
The axial magnetic field strength distribution. (**a**) Ordinary type; (**b**) New type.

We select the cross-section shown in Figure 14 to analyze the distribution of the magnetic field strength axially inside the launcher.

Due to the structural characteristics of the launcher, the magnetic field generated by the rail current cancels each other out at the middle of the launch area, forming a hollow magnetic field, and can meet the requirements of the intelligent load on the magnetic field environment. A strong magnetic field strength appears at the bottom of both armatures, which is related to the distribution of currents. Unlike ordinary type, the new type has a strong magnetic field distribution in the steel rail layer. Compared with the weak magnetic region, it can be found that the hollow area of the new type is larger, indicating that the new type can better meet the electromagnetic shielding requirements.

**Figure 15:**
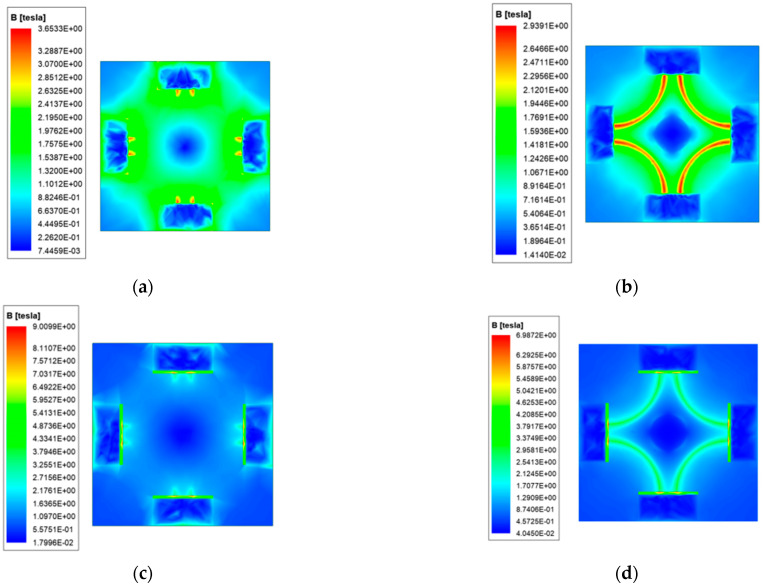
Distribution of magnetic field strength of armature. (**a**) Rear end surface of ordinary type; (**b**) Front end face of ordinary type; (**c**) Rear end surface of new type; (**d**) Front end face of new type.

To analyze the armature magnetic field more intuitively, the magnetic field strength of the front and rear surfaces of the armature is simulated. Figure 15 shows the magnetic field strength distribution cloud diagram of the front and rear surfaces of the armature.

It can be seen that the magnetic field strength distribution of the end surface is symmetrical, and the zero magnetic field area can be clearly seen in the middle area of the armature. The magnetic field strength of the posterior surface is greater than that of the front surface of the armature. The magnetic field distribution of the armature end of the new type is similar to the former, except that the magnetic field strength of the rear surface of the latter can reach up to 9 T, and the front surface can reach 6 T, which is 2.5 times and 2.1 times of the former, respectively. At the same time, there is also a strong magnetic field distribution on steel rail.

**Figure 16:**
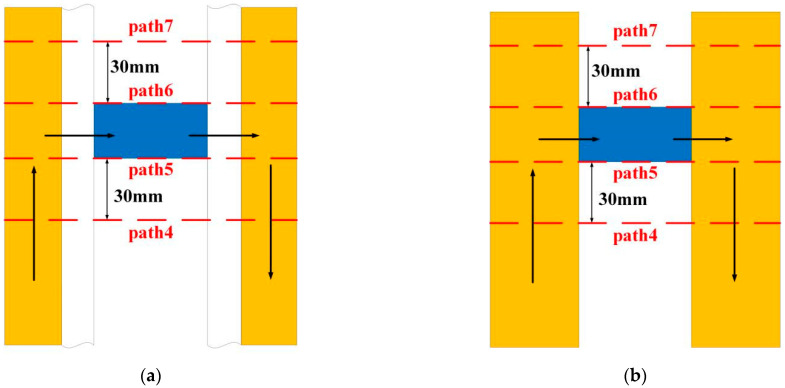
Schematic representation of the four path positions. (**a**) New type; (**b**) Ordinary type.

**Figure 17:**
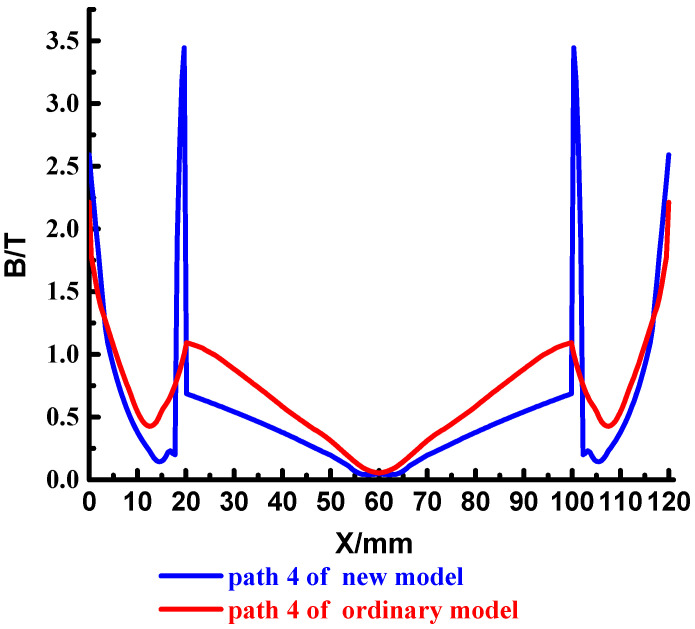
Distribution of the magnetic field strength in path 4.

**Figure 18:**
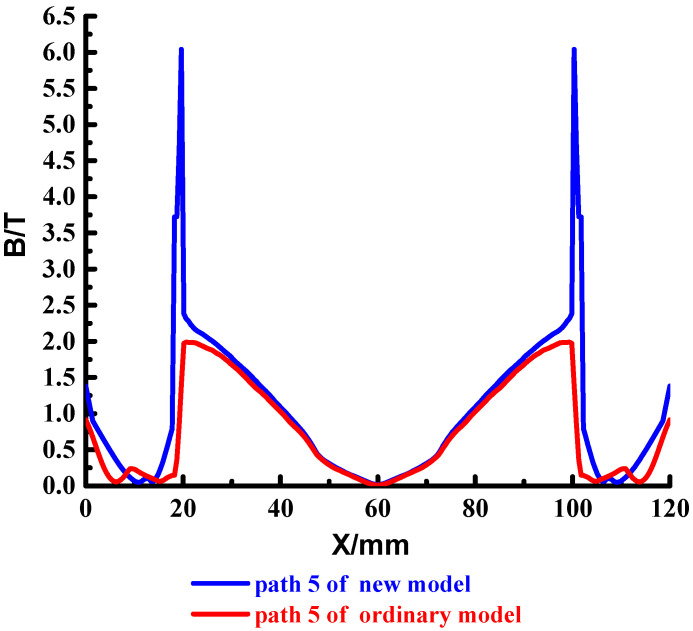
Distribution of the magnetic field strength in path 5.

**Figure 19:**
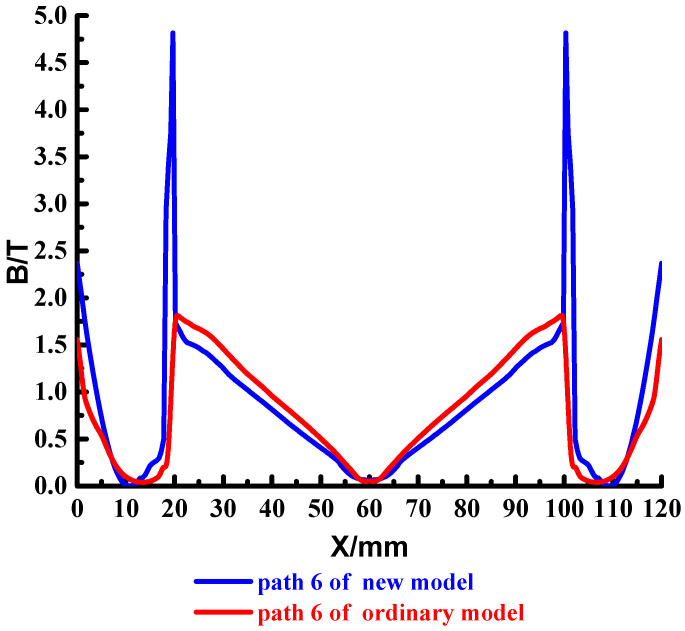
Distribution of the magnetic field strength in path 6.

**Figure 20:**
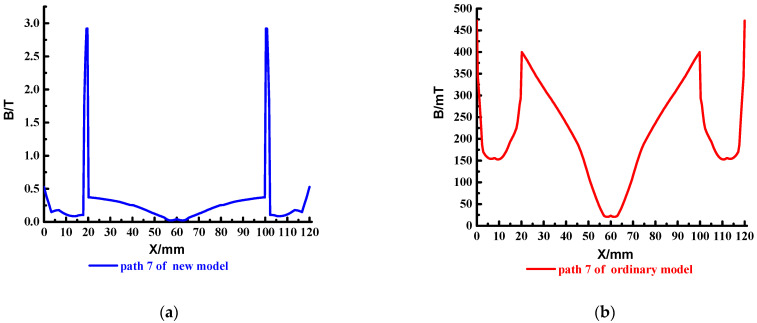
Distribution of the magnetic field strength in path 7. (**a**) New type; (**b**) Ordinary type.

To analyze the magnetic field strength at different locations of the cross-section, set the four paths shown in the red line in Figure 16, which are path 4~path 7. The results are shown in Figure 17, Figure 18, Figure 19 and Figure 20.

The magnetic field strength distribution of the two type on path 4 is similar. Affected by the skin effect of current, the current is mainly distributed on the outside of the rail, the greater the current density, the greater the magnetic field strength excited in space, so the magnetic field strength on the outside of the rail is larger; there is almost no current distribution in the middle of the rail, so the magnetic field strength at that location is very low. At 20 mm, the magnetic field strength has a more obvious increase, this is because due to the influence of the current proximity effect, there is also a certain current distribution on the inside of the rail, which will also stimulate the corresponding magnetic field. The magnetic field, excited by the current, cancels each other out in the launch area, forming a weak magnetic area, realizing electromagnetic self-shielding.

It is worth noting that the magnetic field strength of the copper-steel composite quadrupole rail has a significant mutation at 18 mm (the junction of the copper rail and the steel rail), and the extreme value appears, up to 3.5 T. This is because the permeability of steel is close to 200 times that of air, which is much larger than copper. This is in line with what we analyzed earlier. Comparing the magnetic field strength of the middle magnetic field area of the launch area, it can be seen that the new type is smaller.

The above shows that the magnetic field strength distribution of path 5 and path 6 of the new type and ordinary type is consistent. There is a strong magnetic field strength on the outside of the rail, close to the middle of the rail, the magnetic field gradually decreases, and the magnetic field in the middle of the copper rail drops to 0 T; There is a significant magnetic jump at the copper-steel junction, which gradually decreases in the middle of the launch area.

Compared with the magnetic field strength of the armature front end, the magnetic field strength of the new type is greater than that of the ordinary type, and the weak magnetic field range of the new type is larger than that of the ordinary type, which can better ensure the magnetic field demand of the intelligent load. 

As can be seen from Figure 20, the magnetic field strength of path 7 of ordinary type is very weak, and the maximum is only about 450 mT. The new type has a maximum of 3 T, limited to steel rails, and is less than 0.5 T in most areas.

## 5. Effects of Composite Layer Parameters on Electromagnetic Properties

The above analysis results show that compared with the ordinary type, the electromagnetic characteristics of the launcher will change to a certain extent after the steel layer is added to the rail. To explore the influence of the thickness ratio of copper-steel on the electromagnetic characteristics of the launcher, the thickness of the control rail is 20 mm unchanged, and the launchers with copper-steel thickness ratio of 1:1, 3:1, 4:1, and 9:1 are simulated, the effect of copper-steel thickness ratio on armature and rail current density and magnetic field strength is analyzed, and the relationship between the electromagnetic characteristics of copper-steel composite quadrupole rails and the parameters of the composite layer is studied.

### 5.1. The Influence of the Composite Layer Parameters on the Current Density

**Figure 21:**
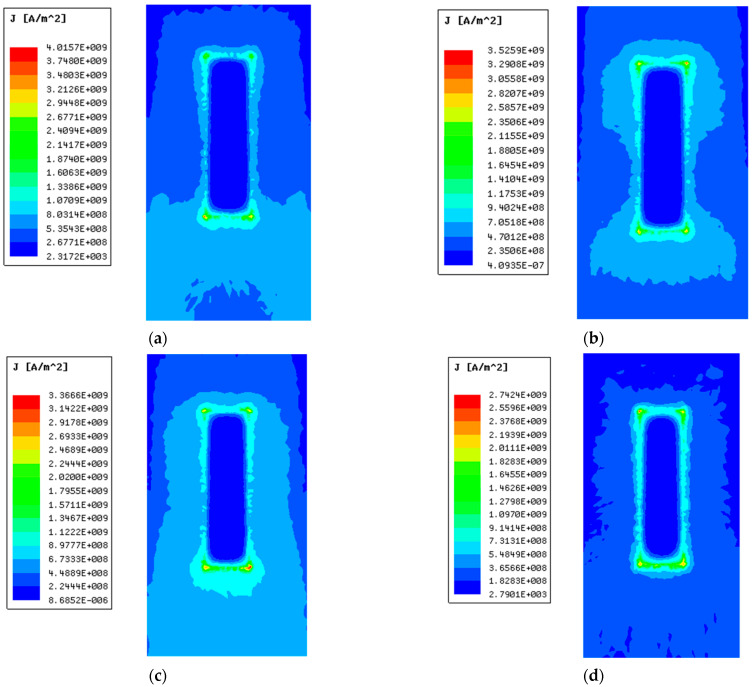
Distribution of current density with different thickness ratios. (**a**) Copper: Steel thickness ratio of 1:1; (**b**) Copper: Steel thickness ratio of 3:1; (**c**) Copper: Steel thickness ratio of 4:1; (**d**) Copper: Steel thickness ratio of 9:1.

Figure 21 shows the current density distribution on the contact surface of the steel rail and armature under different copper-steel thickness ratios.

From Figure 21a–d, it can be seen that the copper-steel thickness ratio mainly affects the current density on the contact surface, and has little impact on the current distribution position. The current is mainly distributed at the edge of the contact surface and the bottom and head of the contact surface, and there is almost no distribution in the middle area of the contact surface. When the copper-steel thickness ratio is 1:1, 3:1, 4:1 and 9:1, respectively, the maximum current density on the steel rail is $4.01\times 10^{9}{A/m}^{2}$, $3.52\times 10^{9}{A/m}^{2}$, $3.36\times 10^{9}{A/m}^{2}$ and $2.74\times 10^{9}{A/m}^{2}$. The copper-to-steel thickness ratio of 9:1 reduces the maximum current density of contact surface by 31.67% compared to the thickness ratio of 1:1. It can be seen that with the increase of the thickness ratio, the maximum current density of the contact surface will decrease, which can alleviate the heat concentration of the contact surface to a certain extent.

Table 3 shows the maximum current density on the contact surface.

Consistent with the above analysis, the maximum current density of the contact surface also decreases with the increase of the thickness ratio. When the copper-steel thickness ratio is 1:1, 3:1, 4:1 and 9:1, respectively, the maximum current density is $3.83\times 10^{9}{A/m}^{2}$, $3.62\times 10^{9}{A/m}^{2}$, $3.06\times 10^{9}{A/m}^{2}$ and $2.25\times 10^{9}{A/m}^{2}$. The copper-steel thickness is 9:1 lower than the maximum current density of the 1:1 armature by 41.2%.

### 5.2. The Influence of the Composite Layer Parameters on the Magnetic Field Strength

**Figure 22:**
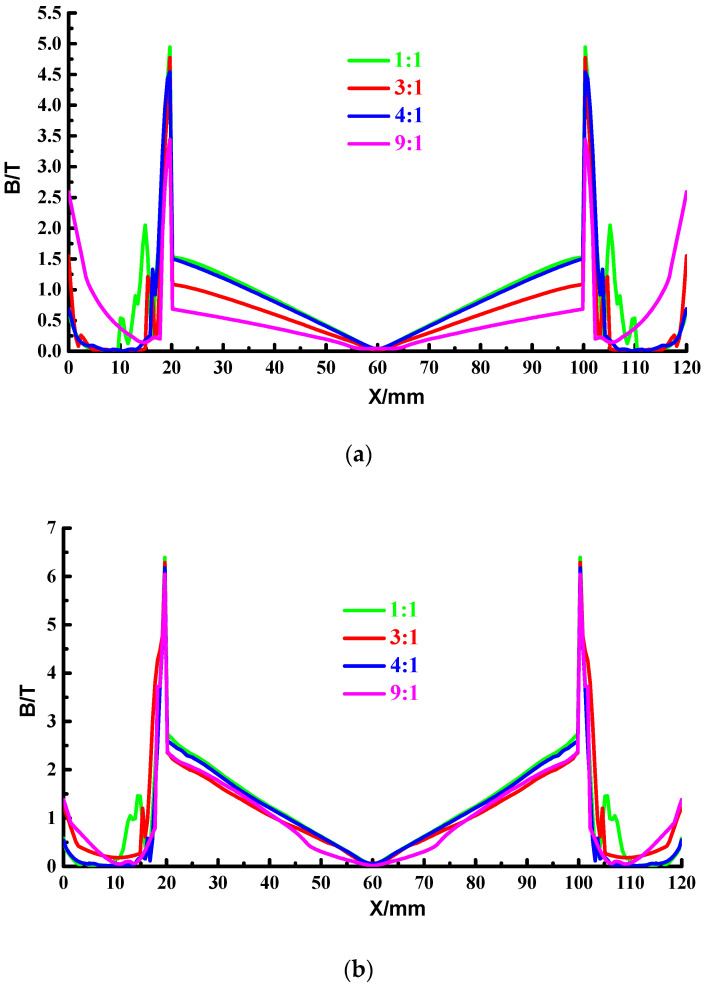

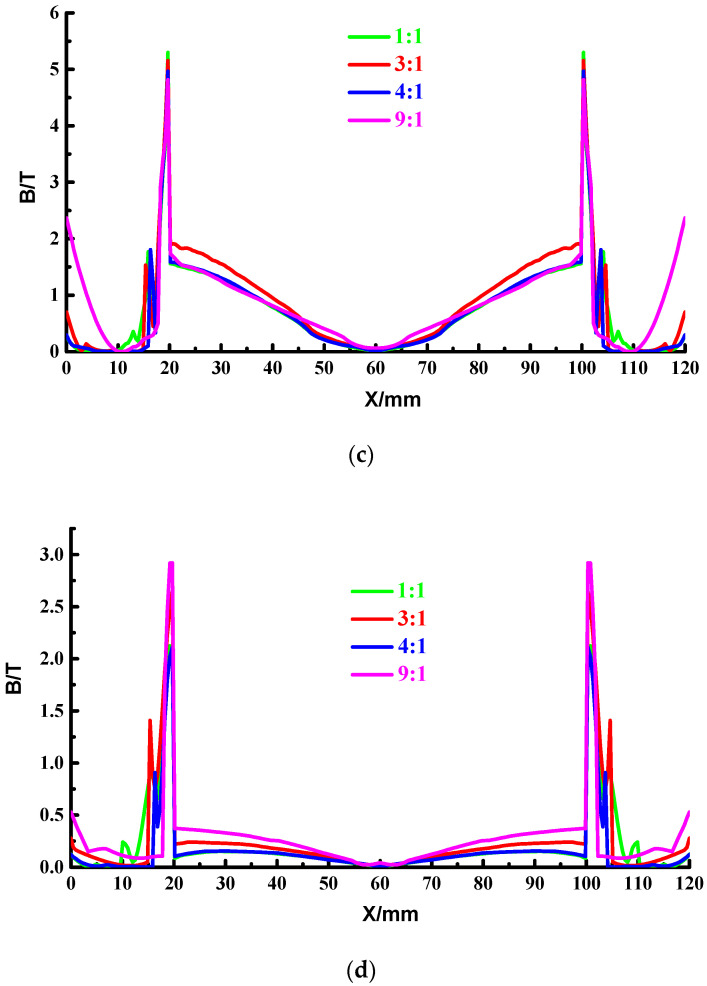
Distribution of magnetic field strength of four paths with different thickness ratios. (**a**) path 4; (**b**) path 5; (**c**) path 6; (**d**) path 7.

To better explore the magnetic field strength distribution characteristics, the magnetic field strength at different locations of the launcher is analyzed, and the four paths shown in Figure 16 are still used. Figure 22 and Table 4 show the maximum magnetic field strength on the four paths under different thickness ratios.

It shows that the magnetic field strength distribution at different thickness ratios is consistent and highly symmetrical. The magnetic field strength distribution is closely related to the current distribution, and the current is more concentrated on the outside of the copper-based rail, so the outside of the copper-based rail has a strong magnetic field strength, while there is almost no current distribution in the middle of the copper-based rail, where the magnetic field strength is weaker and the strength decreases.

Due to the high permeability of the steel, the magnetic field strength at the copper-steel interface increases sharply until it reaches its peak on the inside of the rail. Because the magnetic fields strength generated by the four composite rails in the launch space cancel each other out, the magnetic field strength in the middle of the launch area is weak, and the magnetic field in the middle area of the launch shows a decreasing trend as the thickness of the steel layer increases. It can be seen from Table 4 that the maximum magnetic field strength on each path is negatively correlated with the copper-steel thickness ratio, and the maximum magnetic field strength of the launcher with a thickness ratio of 1:1 is the largest, and the magnetic field strength of the launcher with a thickness ratio of 9:1 is small.

## 6. Conclusions

In this paper, the copper-based composite rail is introduced into the FREL, NFREL is proposed, and the electromagnetic characteristics, such as current density and magnetic field strength, are compared and analyzed. This paper introduces composite materials into the electromagnetic launch rail to solve the problem of the rail life of the FREL and verifies their scientific and rationality, which further promotes electromagnetic launch technology from the laboratory to engineering applications. Through the analysis, it can be found that:

(1) The current density of contact surface of the NFREL is significantly reduced, the zero area of the contact surface is larger, and the heat production decreases, indicating that the composite rail can effectively alleviate the thermal damage of the rail and improve the current distribution on the contact surface; The introduction of steel layer rails can increase the wear resistance of the rail and prolong the life of the rail.

(2) The hollow area of the magnetic field of the NFREL is larger, which can provide a good magnetic field launch environment and better meet the electromagnetic shielding requirements.

(3) The copper-steel thickness ratio will have a certain impact on the armature and rail current density and magnetic field strength, and attention should be paid to choosing the appropriate copper-steel thickness ratio.

In this paper, only the electromagnetic characteristics of the NFREL are simulated and analyzed, and the other characteristics of the rail of the NFREL, such as the vibration characteristics, were not analyzed in depth, which will also be the focus of subsequent research to this paper.

## Figures and Tables

**Table 1:** Armature and rail material properties.

	Density/( ${\mathrm{Kg}/m}^{-3}$)	Conductivity/(S/m)	Relative Magnetic Permeability
Copper rail	8900	$5.8\times 10^{7}$	1
Steel rail	7800	$2.0\times 10^{6}$	200
Aluminum armature	2700	$3.8\times 10^{7}$	1

**Table 2:** Grid Parameters and Verification.

	The First Calculation	The Second Calculation	The Third Calculation
Maximum grid size (mm)	0.5	1	2
maximum current density ($\times 10^{9}{A/m}^{2}$)	7.48	7.28	6.86

**Table 3:** Maximum current density with composite layer parameters.

	Copper-Steel Thickness Ratio			
	1:1	3:1	4:1	9:1
$I_{\max}$ $/(\times 10^{9}A/m^{2})$	3.8	3.6	3.0	2.25

**Table 4:** Maximum magnetic field strength with composite layer parameters.

		Copper-Steel Thickness Ratio			
		1:1	3:1	4:1	9:1
$B_{\max}$ /(T)	path 4	4.91	4.72	4.51	3.44
	path 5	6.29	6.24	6.17	6.04
	path 6	5.33	5.15	4.98	4.86
	path 7	3.12	3.07	2.96	2.92
